# 2D Ising Model for Enantiomer Adsorption on Achiral Surfaces: L- and D-Aspartic Acid on Cu(111)

**DOI:** 10.3390/e24040565

**Published:** 2022-04-18

**Authors:** Soham Dutta, Andrew J. Gellman

**Affiliations:** 1 Department of Chemical Engineering, Carnegie Mellon University, Pittsburgh, PA 15213, USA; sohamd@andrew.cmu.edu; 2 Wilton E. Scott Institute for Energy Innovation, Carnegie Mellon University, Pittsburgh, PA 15213, USA

**Keywords:** Ising model, adsorption, chirality, enantiomers, symmetry breaking

## Abstract

The 2D Ising model is well-formulated to address problems in adsorption thermodynamics. It is particularly well-suited to describing the adsorption isotherms predicting the surface enantiomeric excess, $ee_{s}$, observed during competitive co-adsorption of enantiomers onto achiral surfaces. Herein, we make the direct one-to-one correspondence between the 2D Ising model Hamiltonian and the Hamiltonian used to describe competitive enantiomer adsorption on achiral surfaces. We then demonstrate that adsorption from racemic mixtures of enantiomers and adsorption of prochiral molecules are directly analogous to the Ising model with no applied magnetic field, i.e., the enantiomeric excess on chiral surfaces can be predicted using Onsager’s solution to the 2D Ising model. The implication is that enantiomeric purity on the surface can be achieved during equilibrium exposure of prochiral compounds or racemic mixtures of enantiomers to achiral surfaces.

## 1. Introduction

**Figure 1:**
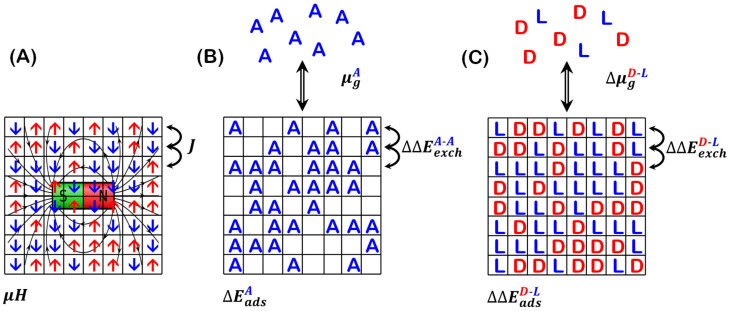
(**A**) Illustration of the 2D Ising model for spins interacting through nearest neighbor exchange interaction, J, in the presence of an applied magnetic field, $\vec{H}$. (**B**) Equivalent illustration for adsorbate, A, with adsorption energy, $\Delta E_{ads}^{A}$, from a gas phase with chemical potential, $\mu_{g}^{A}$, onto a square lattice of adsorption sites. The interaction $\Delta \Delta E_{exch}^{\text{A-A}}$ occurs between A ’s adsorbed on adjacent nearest neighbor sites. (**C**) Illustration of enantiomer adsorption at saturation coverage. The difference in adsorption energies is $\Delta \Delta E_{ads}^{\text{D-L}}=0$ on an achiral surface. The adsorbate–adsorbate interaction is quantified by the difference in energy between homochiral pairs and heterochiral pairs of adjacent adsorbates. Figure 1A,C are reprinted/adapted with permission from [5]. Copyright 2020, John Wiley and Sons.

There is a historical link between the 2D Ising model and the adsorption isotherms describing atoms and molecules adsorbing from the gas phase onto surfaces [1,2]. The Ising model was originally conceived to describe the magnitude of spin polarization (induced magnetism) in ferromagnetic materials subjected to an external applied magnetic field [3,4]. In the field of surface chemistry, the adsorption isotherm is used to describe the coverages of adsorbed molecules as a function of gas phase partial pressures at a fixed temperature. The conceptual connection between these phenomena and their associated models is made clear by the illustrations of the two shown in Figure 1A,B. Recently, it has been shown that the competitive adsorption of the enantiomers of a chiral molecule is amenable to description by the 2D Ising model [5]. In addition, the 2D Ising model predicts that at low temperatures, the adsorption of prochiral molecules can lead to enantiomerically pure adsorbed monolayers.

The 2D Ising model describes a system consisting of a set of spins distributed on the sites of a 2D square lattice, such that there is one spin per lattice point. The spins are oriented either up or down and the spins at the lattice points i=1…N are given by the vector $\vec{S}=\left(s_{1}\;\ldots \;s_{N}\right)$, where $s_{i}=1$ indicates ↑ and $s_{i}=-1$ indicates ↓. The spins interact with an externally applied magnetic field, $\vec{H}$, such that the associated energy is given by $\mu Hs_{i}$ where μ is magnetic moment. In addition, adjacent spins interact with an energy given by $s_{i}s_{j}J$, where site j must be adjacent to site i and J is an exchange or interaction energy. The Hamiltonian for the energy of this system is given by:

(1) $$ℋ\left(\vec{S}\right)=-\mu H\sum_{i=1}^{N}s_{i}-\frac{1}{2}\;J\sum_{i=1}^{N}\left(s_{i}\sum_{nn_{i}=1}^{4}s_{nn_{i}}\right)$$

where the sum over $nn_{i}$ is summing over the four nearest neighbor sites of site i. The summation in the first term yields the difference in the number of spin-up versus spin-down sites. The summations in the second term yield the difference in the number of spin-aligned versus spin-misaligned nearest neighbor pairs.

The adsorption problem is typically described by a surface with an array of discrete equivalent adsorption sites on which molecules in the gas phase can adsorb. At a given temperature, the adsorbed molecules are in equilibrium with the gas phase pressure, and the fractional occupation of adsorption sites, θ(P;T), is the isotherm that describes the system. Sites may be occupied or empty. In this case, the adsorption energy, $\Delta E_{ads}^{A}$, takes the role played by the magnetic field, $\vec{H}$. The adsorbate–adsorbate nearest neighbor interaction energy, $\Delta E_{exch}^{\text{A-A}}$, is the *A-A* interaction energy needed to separate two adjacent adsorbates. This plays the role of the exchange constant J in the Ising model. When adsorbates are non-interacting, i.e. $\Delta E_{exch}^{\text{A-A}}=0$, this model equates with the first-order Langmuir adsorption isotherm.

## 2. Langmuir Adsorption Isotherms

The simplest of the many models for adsorption onto surfaces is attributed to Langmuir [6] and can be regarded as the ‘ideal’ adsorption isotherm in the sense that it describes non-interacting adsorbates. The Langmuir model for adsorption on a surface describes systems in which: all adsorption sites are equivalent, adsorbates are non-interacting, and there can be only one or zero adsorbates per site. Under these constraints, the adsorption isotherm for molecules that adsorb non-dissociatively, i.e., with first-order adsorption and desorption kinetics, is given by:

(2) $$\theta_{A}\left(P_{A};T\right)=\frac{K_{A}P_{A}}{1+K_{A}P_{A}}\;$$

where $P_{A}$ is the gas phase partial pressure of component A. The temperature dependence of the coverage is dictated by the adsorption equilibrium constant, $K_{A}$. In the limit that $K_{A}P_{A}\to \infty$, the coverage is $\theta_{A}\approx 1$ and the surface is saturated with adsorbate.

The Langmuir model also describes competitive adsorption between non-interacting adsorbates A and B on a surface. The isotherms in this case are:

(3a) $$\theta_{A}\left(P_{A},P_{B};T\right)=\frac{K_{A}P_{A}}{1+K_{A}P_{A}+K_{B}P_{B}}\;$$

(3b) $$\theta_{B}\left(P_{A},P_{B};T\right)=\frac{K_{B}P_{B}}{1+K_{A}P_{A}+K_{B}P_{B}}\;$$

Competitive adsorption is not amenable to description by the Ising model because the site descriptors would take three values: occupied by A, by B, or vacant. However, the case in which the surface is saturated, $\theta_{A}+\theta_{B}=1$, is described by the Ising model because there are no vacant sites. In this case, the differences in the adsorption energies, $\Delta \Delta E_{ads}^{A-B}=\Delta E_{ads}^{A}-\Delta E_{ads}^{B}$, and the difference in the gas phase chemical potentials, $\Delta \mu_{g}^{A-B}=\mu_{g}^{A}-\mu_{g}^{B}$, are equivalent to the applied magnetic field.

The problem of enantiomer adsorption on surfaces is an interesting special case of competitive co-adsorption (Figure 1C). Enantiomers are the two non-superimposable mirror images of a chiral molecule. They are typically labeled as the D- and L-enantiomer, for ‘dextro’ and ‘levo’, as per some convention for denoting one enantiomer as right-handed and the other as left-handed. The relevant adsorption problem is to relate the enantiopurity of adsorbed enantiomer mixtures to the enantiopurity of the gas phase mixture. Enantiopurity is typically quantified in terms of enantiomeric excess, ee. In the gas phase, enantiomeric excess is defined as $ee_{g}=\frac{P_{D}-P_{L}}{P_{D}+P_{L}}$. The equimolar enantiomer mixture with $ee_{g}=0$ is referred to as ‘racemic’. On a saturated surface, the enantiomeric excess is given by $ee_{s}=\frac{\theta_{D}-\theta_{L}}{\theta_{D}+\theta_{L}}=\theta_{D}-\theta_{L}$, where $ee_{s}$ is equivalent to the induced magnetization in the Ising model for spins [4,5]. Note that a positive value of ee corresponds to an excess of the D-enantiomer.

On achiral surfaces, symmetry dictates that the adsorption energies of the two enantiomers are identical, $\Delta E_{ads}^{D}=\Delta E_{ads}^{L}$. In the Langmuir model for enantiomer adsorption onto an achiral surface, this would lead to the enantiomer adsorption equilibrium constants being equal, $K_{D}=K_{L}$. In the presence of a gas phase mixture of enantiomers at a total pressure, $P_{tot}=P_{D}+P_{L}$, sufficient to yield saturation coverage, $\theta_{D}+\theta_{L}=1$, Equation (3a,b) yields $ee_{s}=ee_{g}$. In practice, this is not observed [5,7]. The critical missing feature in the Langmuir model is the accounting for adsorbate–adsorbate interactions between enantiomers. There are a variety of empirical models for adsorption isotherms that account for these inter-adsorbate interactions in different ways [2]. In the framework of the Ising model, these are accounted for by the difference in the interactions between homochiral and heterochiral nearest neighbors, $\Delta \Delta E_{exch}^{D-L}$, which serves the role of the exchange coupling constant, J.

## 3. Enantiomer Co-Adsorption: D- and L-Asp on Cu(111)

**Figure 2:**
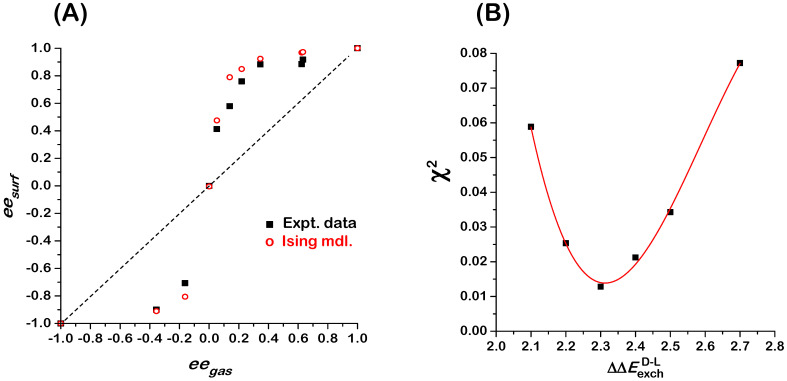
(**A**) Plot of $ee_{s}$ versus $ee_{g}$ for gas phase mixtures of D- and L-Asp in equilibrium with Asp adsorbed on the Cu(111) surface at 460 K: experimental measurements (solid black squares), predictions of Monte Carlo simulations using the 2D Ising model and a 100 × 100 square lattice (open red circles). (**B**) Plot of the residual, $\chi^{2}$, arising from fitting the results of the 100 × 100 Monte Carlo simulation obtained using values of $\Delta \Delta E_{exch}^{\text{D-L}}$ spanning the range 2.1 to 2.7 kJ/mole. The red line is a fit of a cubic polynomial to the values of $\chi^{2}$, showing the minimum at $\Delta \Delta E_{exch}^{\text{D-L}}=2.31$ kJ/mole. Data in Figure 2A are reproduced from [7].

We have measured $ee_{s}$ versus $ee_{g}$ for competitive co-adsorption of amino acid enantiomer mixtures (alanine, proline, and aspartic acid) on chiral and achiral Cu(*hkl*) single-crystal surfaces [7,8,9,10,11]. Herein, we focus on the results obtained using aspartic acid (D- and L-Asp) adsorption onto the achiral Cu(111) surface (Figure 2A). The details of the experimental method have been described elsewhere [7,10]. Briefly, the experiments using the Cu(111) single crystal were conducted in an ultra-high vacuum chamber, in which the clean Cu(111) surface at 460 K was exposed to gas phase mixtures of D- and L-Asp sublimated from independent Knudsen cells with controlled $ee_{g}$. Following saturation of the surface and the establishment of equilibrium adsorption, the value of $ee_{s}$ was measured by thermally decomposing the Asp monolayer and using mass spectrometry to quantify the yields of ^13^CO_2_ from 1,4-^13^C_2_-L-Asp and ^12^CO_2_ from D-Asp.

The data for competitive enantiomer adsorption in Figure 2A reveal a clear deviation from Langmuir-like behavior. Whereas the Langmuir isotherm for competitive enantiomer adsorption predicts that $ee_{s}=ee_{g}$, we observe an auto-amplification of enantiomeric excess in which $\left|ee_{s}\right|\geq \left|ee_{g}\right|$ [7]. In other words, the act of adsorption on an achiral surface can lead to enantiopurification of a non-racemic mixture, even on an achiral surface. The equality holds true for $ee_{g}=0,\pm 1$, as expected. When starting with an enantiomerically pure gas phase, one observes an enantiomerically pure adsorbed phase. When the gas phase is achiral and the surface is also achiral, it seems reasonable that the adsorbed phase should be racemic (as observed), but as the Ising model demonstrates, that is not necessarily true. Similar amplification of enantiomeric excess, as indicated by the positive deviations of $\left|ee_{s}\right|$ relative to $\left|ee_{g}\right|$, has been observed in several other systems of amino acid adsorption on Cu surfaces: Asp/Cu(643)^R&S^ [8], Asp/Cu(653)^R&S^ [11], and Pro/Cu(643)^R&S^ [9].

The open red circles in Figure 2A are the predictions of Monte Carlo simulations of the 2D Ising model on a 100 × 100 square lattice with periodic boundary conditions [5]. The Hamiltonian used for these simulations is expressed as:

(4) $$ℋ\left(\vec{Χ}\right)=-\left(\Delta \Delta E_{ads}^{\text{D-L}}+RT\cdot \Delta \mu_{g}^{\text{D-L}}\right)\sum_{i=1}^{N\;}\chi_{i}-\frac{1}{2}\Delta \Delta E_{exch}^{\text{D-L}}\sum_{i=1}^{N\;}\left(\chi_{i}\sum_{nn_{i}=1}^{4\;}\chi_{nn_{i}}\right)$$

where $\chi_{i}=\pm 1$ enumerates the chirality (D- or L-) of the adsorbate at site i and the lattice is constrained to be saturated with enantiomeric adsorbates. The summation in the first term yields the difference between the numbers of D- and L-enantiomers adsorbed on the surface. The summations in the second term yield the difference in the number of homochiral versus heterochiral nearest neighbor pairs on the surface. The direct analogy between the description of a system of interacting spins and a system of enantiomers competing for adsorption sites is clear from the comparison of Equations (1) and (4). In the adsorption problem, there are two driving forces that play the role of the magnetic field applied to the spin system. The first is the difference in the enantiomer adsorption energies, $\Delta \Delta E_{ads}^{\text{D-L}}=\Delta E_{ads}^{D}-\Delta E_{ads}^{L}$. This is $\Delta \Delta E_{ads}^{\text{D-L}}=0$ on an achiral surface such as Cu(111). On chiral surfaces, $\Delta \Delta E_{ads}^{\text{D-L}}\neq 0$ has been observed in a number of systems [10,12]. The second driving force taking the role of the applied magnetic field is the difference in the chemical potentials of the two enantiomers in the gas phase, $\Delta \mu_{g}^{\text{D-L}}$. Under UHV conditions used for the measurements in Figure 2, $\Delta \mu_{g}^{\text{D-L}}=lnP_{D}/P_{L}=ln\left(\frac{1+ee_{g}}{1-ee_{g}}\right)$.

Monte Carlo simulations using the 2D Ising model for competitive D- and L-Asp enantiomer adsorption on the Cu(111) surface were conducted at the same values of $ee_{g}$ as were used for our measurements of $ee_{s}$ shown in Figure 2A (black squares). The simulations were conducted using a temperature of 460 K (same as experiments), values of $\Delta \Delta E_{exch}^{\text{D-L}}=$ 2.3 kJ/mole for the exchange interaction energy, $\Delta \Delta E_{ads}^{\text{D-L}}=$ 0 for the adsorption energy difference, and values of $\Delta \mu_{g}^{\text{D-L}}$ consistent with the values of $ee_{g}$ used experimentally. It is clear that the 2D Ising model (open red circles) reproduces the behavior observed experimentally. 

The experimental data in Figure 2A have been used to estimate the magnitude of the exchange energy, $\Delta \Delta E_{exch}^{\text{D-L}}$, between D- and L-Asp on Cu(111). Monte Carlo simulations of $ee_{s}$ versus $ee_{g}$ were conducted using the parameters listed in the previous paragraph, but with values of $\Delta \Delta E_{exch}^{\text{D-L}}$ spanning the range 2.1 to 2.7 kJ/mol. At each value of $\Delta \Delta E_{exch}^{\text{D-L}}$, the residual deviation, $\chi^{2}$, between the Monte Carlo results and the experimental data has been evaluated and plotted in Figure 2B (solid squares). The fit of a cubic polynomial to the values of $\chi^{2}$ indicates that the best fit to the data occurs for $\Delta \Delta E_{exch}^{\text{D-L}}=2.31$ kJ/mole. The positive value of $\Delta \Delta E_{exch}^{\text{D-L}}$ indicates that homochiral D-D and L-L nearest neighbor interactions are more attractive than heterochiral D-L nearest neighbor interactions for the Asp/Cu(111) system. The dominant homochiral interactions lead to the amplification of enantiomeric excess on the surface relative to the gas phase. If the heterochiral interactions were dominant, i.e., $\Delta \Delta E_{exch}^{\text{D-L}}<0$, then the enantiomeric excess on the surface would be suppressed relative to the gas phase and the monolayer composition would tend towards racemic.

**Figure 3:**
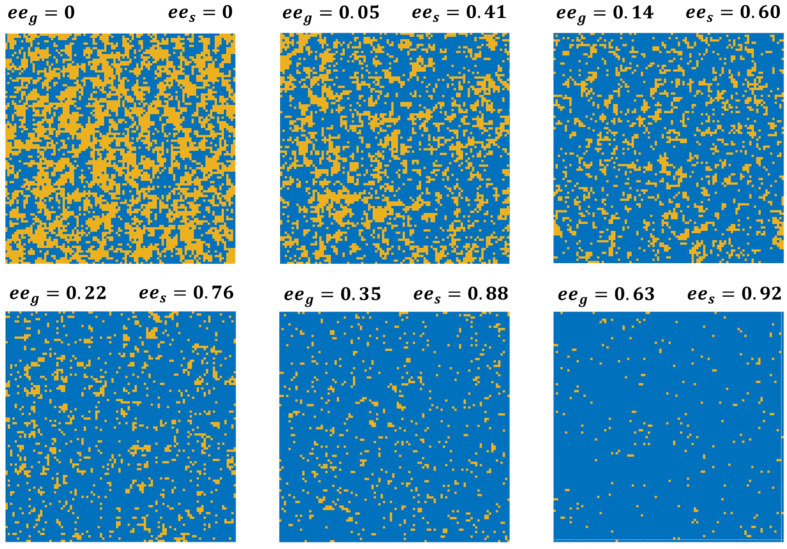
Illustrations of the enantiomer distributions on the 100 × 100 square lattice used for 2D Ising model simulation of competitive enantiomer adsorption. Blue sites are occupied by D-enantiomers and orange sites are occupied by L-enantiomers. Simulations were conducted using *T* = 460 K, $\Delta \Delta E_{exch}^{\text{D-L}}=2.31\;\mathrm{kJ}/\mathrm{mol}$, and values of $ee_{g}$ in the range of 0 to 0.63. The values of $ee_{g}$ and resulting $ee_{s}$ are shown for each MC simulation.

Representative maps of the lattice occupation obtained from equilibrated simulations of the 2D Ising model are shown in Figure 3 for six of the values of $ee_{g}$ at which equilibrium measurements of $ee_{s}$ have been performed and reported in Figure 2B. These simulations have been conducted using $\Delta \Delta E_{exch}^{\text{D-L}}=2.31$ kJ/mole, the value that yields the best fit to the experimental data in Figure 2A. Even when $ee_{g}=0$ and results in $ee_{s}=0$, the effect of the positive value of $\Delta \Delta E_{exch}^{\text{D-L}}$ can be observed in the form of homochiral clustering on the surface. The driving force to increase the number of homochiral interactions leads to further clustering at $ee_{g}=0.05$, such that the value of $ee_{s}$ is almost an order of magnitude higher than $ee_{g}$.

## 4. Implications of the 2D Ising Model for Enantiomer and Prochiral Adsorption

The Hamiltonian (Equation (4)) used as the basis for modeling competitive D- and L-Asp enantiomer adsorption on Cu(111) reproduces the sigmoidal shape of the experimental data in Figure 2, including the value of $ee_{s}=0$ for a racemic mixture in the gas phase, $ee_{g}=0$. Note that for chiral surfaces with $\Delta \Delta E_{ads}^{\text{D-L}}\neq 0$, competitive enantiomer adsorption experiments reveal a non-zero value of $ee_{s}$ for a racemic mixture, $ee_{g}=0$, in the gas phase [10]. This is because the chiral surface has an affinity for adsorption of one enantiomer over the other. However, the Ising model also predicts the possibility of achieving $ee_{s}\neq 0$ from a racemic mixture in the gas phase during low-temperature adsorption.

The constraints that the surface be achiral, $\Delta \Delta E_{ads}^{\text{D-L}}=0$, and the gas phase be racemic, $\Delta \mu_{g}^{\text{D-L}}=0$, are equivalent to the absence of an applied magnetic field, $\vec{H}=0$, in the Ising description of spin systems. This is a special case of the 2D Ising model that has an analytical solution derived by Lars Onsager [13] for the magnitude of the spin polarization, or in our case, the enantiomeric excess on the surface [4].

(5) ees=[1−sinh−4(ΔΔEexchD-LRT)]1/8

In the absence of other driving forces, $\Delta \Delta E_{exch}^{\text{D-L}}$ dictates the $ee_{s}$ of the adsorbed monolayer in equilibrium with a racemic mixture in the gas phase. When $\Delta \Delta E_{exch}^{\text{D-L}}<0$ and heterochiral interactions are favored over homochiral interactions, the adsorbed monolayer will be racemic. On the other hand, when homochiral interactions are favored, $\Delta \Delta E_{exch}^{\text{D-L}}>0$, Onsager’s equation (Equation (5)) predicts the formation of chiral monolayers at low temperatures. Onsager’s equation predicts the existence of a phase transition at a critical temperature, $T_{c}$, defined by:

(6) $$RT_{c}=\Delta \Delta E_{exch}^{\text{D-L}}/ln\left(1+\sqrt{2}\right)$$

At temperatures $T>T_{c}$, the Onsager solution predicts that $ee_{s}=0$, as observed in the simulations and experiments (Figure 2A). On the other hand, at $T<T_{c}$, the Onsager solution predicts the formation of an adsorbed monolayer with chirality approaching $\left|ee_{s}\right|\approx 1$ as the temperature decreases. Note that the sign of $ee_{s}$ is not determined and, in the absence of a chiral driving force, one is equally likely to find $ee_{s}\approx \pm 1$.

The predictions of Onsager’s solution to the 2D Ising model for enantiomer adsorption are quite remarkable from the perspective of conducting enantiomer separations via adsorption processes. The implication is that for enantiomers that exhibit homochiral attractions even on surfaces that have no intrinsic chirality, one could achieve arbitrarily high enantiopurity, provided that the adsorption temperature is $T<T_{c}$. It is worth pointing out that with an exchange energy of $\Delta \Delta E_{exch}^{\text{D-L}}=2.31$ kJ/mole, the critical temperature is $T_{c}≅315$ K. In contrast, our experimental adsorption temperature was significantly higher at T=460 K, so neither our experiment nor the Monte Carlo simulations should be expected to exhibit this phase transition yielding enantiospecific adsorption from a racemic gas phase mixture. Our simulations to quantify $\Delta \Delta E_{exch}^{\text{D-L}}$ yield an accurate estimate of $T_{c}=315\;K$. This will guide future experiments aimed at observing enantiomer purification on achiral surfaces exposed to gas phase mixtures with $ee_{g}=0$ and $T<T_{c}$. 

**Figure 4:**
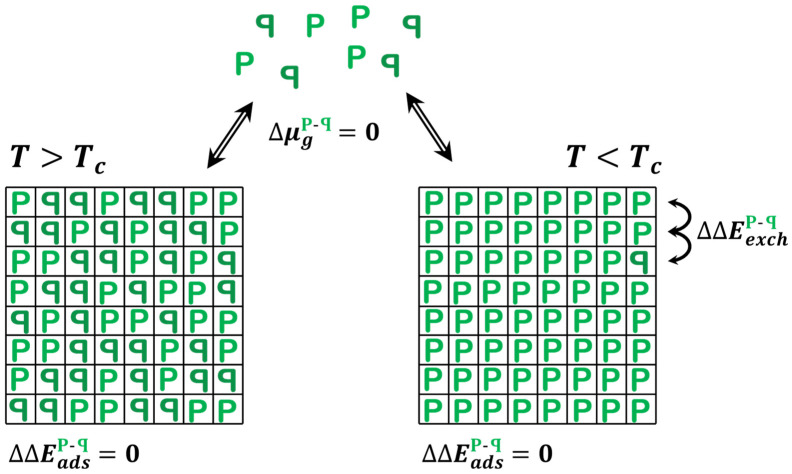
Illustration of the 2D Ising model predictions for equilibrium adsorption of prochiral molecules onto an achiral surface at $T>T_{c}$ and $T<T_{c}$. Note that P and ꟼ are achiral and truly identical in the gas phase because they are free to rotate out of the plane of the page. That degree of freedom is frozen out in the adsorbed state, although the molecules can ‘flip’ between states, but not freely, i.e., there is a barrier to flipping. The 2D Ising model predicts that for $T<T_{c}$, the equilibrium adsorbed state can be arbitrarily close to homochiral. Figure 4 reprinted/adapted with permission from [5]. Copyright 2020, John Wiley and Sons.

There is a related adsorption system that is also ideally represented by the 2D Ising model: the adsorption of prochiral molecules onto achiral surfaces (Figure 4). Prochiral molecules are achiral in the gas phase but are rendered chiral upon adsorption. The simplest example is a molecule or object with one plane of mirror symmetry—consider the object ‘P’. In 3D, such objects can rotate freely such that P and ꟼ are identical; however, once adsorbed on a surface, the two configurations are enantiomers of one another. The surface breaks the mirror plane symmetry. On achiral surfaces, the enantiomer adsorption energies are identical, $\Delta \Delta E_{ads}^{\mathrm{P-ꟼ}}=0$. Furthermore, since there is only one species in the gas phase, there is no shift in the gas phase chemical potential associated with a change in the relative concentrations of enantiomers, $\Delta \mu_{g}^{\mathrm{P-ꟼ}}=0$. In fact, it does not really make sense to define the quantity $\Delta \mu_{g}^{\mathrm{P-ꟼ}}$ because P*_g_* and ꟼ*_g_* are neither chiral nor enantiomers of one another in the gas phase. This renders the model for equilibrium adsorption of prochiral molecules onto achiral surfaces exactly equivalent to the case of the 2D Ising model for which Onsager’s solution applies. In fact, one need not consider adsorption at all, because the interconversion between P and ꟼ can be achieved simply by literally flipping the molecule over to change configurations on the surface. For prochiral adsorbates with $\Delta \Delta E_{exch}^{\mathrm{P-ꟼ}}>0$ and conditions $T<T_{c}$, the prochiral gas phase species will form an adsorbed monolayer with arbitrarily high $ee_{s}$.

The key point of this work is that symmetry breaking to yield an enantiomerically pure monolayer from a racemic mixture on an achiral surface can occur as the result of a reversible thermodynamic process. Chiral amplification has also been observed during adsorption of prochiral propylene on an achiral Pt(111) surface [14,15]. However, that surface was first seeded with low coverages of chiral R- or S-propylene oxide, rendering the adsorbing surface chiral prior to exposure and adsorption of the propylene. Moreover, the origin of the chiral amplification was deemed to originate with enantiospecific adsorption kinetics. This was successfully corroborated using kinetic Monte Carlo models, but ones that are significantly more complex than the 2D Ising model describing the adsorption equilibrium that is the focus of this study.

## 5. Implications of the 2D Ising Model for the Origins of Homochirality in Life

One of the most important societal consequences of molecular chirality arises from the fact that the biomolecules (DNA, proteins, sugars, etc.) on which life is based are all chiral but present only in one enantiomeric form in living organisms. The consequence is that chiral pharmaceuticals and other bioactive compounds must be prepared and administered in enantiomerically pure form. This motivates the need to develop enantioselective chemical processes such as catalysis and adsorption for use in the pharmaceutical and bio chemical industries. These can be achieved using chiral surfaces. However, by understanding competitive enantiomer adsorption in terms of the 2D Ising model, it becomes clear that enantiomer purification can also be achieved using achiral surfaces.

The origin of homochirality in life on Earth is one of those mysteries that has been and will continue to be a subject of speculation [16,17,18]. Assuming that the primordial soup from which life evolved started as an achiral medium, some form of symmetry breaking occurred that led to homochirality. It may be the case that early forms of life were based on racemic compounds, but that biochemistry led to the formation of chiral compounds which, in homochiral form, provided an evolutionary advantage.

It is also the case that there are physical (non-biological) processes that can lead to symmetry breaking and could have predated life on Earth, leading to local enantiomeric enrichment of chiral compounds or materials. If the subsequent appearance of early life occurred in a zone of enantiomeric enrichment, there would have been a chiral bias present to seed the homochirality of life. The earliest physical model for spontaneous symmetry breaking leading to homochirality was proposed by Frank and invokes chiral autocatalysis [19]. The initial step in Frank’s model is the slow spontaneous conversion of an achiral species A into a chiral product, A→D (or L), where each product enantiomer is equally likely. The second step is the further rapid conversion of the initial reactant, A, into a chiral product via an autocatalytic and enantioselective process involving the chiral product as the catalyst, $A\underset{D\left(L\right)}{\to }D\;\left(\mathrm{or}\;L\right)$. This leads to an exponential growth in the chiral product concentration. However, if there is a slight excess of one enantiomer over the other, as a result of statistical fluctuation in yield, and there is a third process by which heterochiral dimerization of the product into a non-catalytic species occurs, DL, the minority species will be sequestered while the majority enantiomer increases in concentration until the reactant is fully consumed. Even small statistical fluctuations in the early product enantiomer concentration could have been sufficient to yield a final product with arbitrarily high enantiomeric excess.

One of the important features of the work discussed herein is the understanding that the simple process of chiral or prochiral species adsorption from a gas or liquid phase onto an achiral surface is sufficient to lead to locally high enantiopurity. The Frank mechanism for spontaneous symmetry breaking is fundamentally based in reaction kinetics and the key features that it invokes are enantiospecific autocatalysis coupled with an irreversible product sequestration step. In contrast, the 2D Ising model discussed in this work is a purely thermodynamic model that allows achiral systems to evolve to homochirality through purely statistical fluctuations.

## Data Availability

Data is available by contacting the corresponding author.
